# Development and Temporal Validation of a Three-Metabolite Cerebrospinal Fluid Model for the Diagnosis of Leptomeningeal Metastasis from Solid Tumors

**DOI:** 10.3390/diagnostics16203316

**Published:** 2026-10-12

**Authors:** Huaixu Li, Hongqing Cai, Shen Tian, Yujia Chen, Qi Liu, Jinghai Wan

**Affiliations:** Department of Neurosurgery, National Cancer Center/National Clinical Research Center for Cancer/Cancer Hospital, Chinese Academy of Medical Sciences and Peking Union Medical College, Beijing 100021, China; lihuaixu1997@163.com (H.L.);

**Keywords:** leptomeningeal metastasis, cerebrospinal fluid, targeted metabolomics, liquid chromatography–mass spectrometry, diagnostic model, internal validation

## Abstract

**Background/Objectives**: Leptomeningeal metastasis (LM) is a severe complication of solid tumors. Cerebrospinal fluid (CSF) cytology is only about 70% sensitive on a single examination, and objective markers are needed. **Methods:** CSF from 49 patients with LM and 16 with intracranial disease without LM was analyzed by targeted LC–MS in a training batch (*n* = 30) and a temporally independent validation batch (*n* = 35). Metabolites selected by both LASSO and random forest entered a logistic regression model, which was applied unchanged to the validation batch and evaluated by repeated and nested cross-validation on all 65 samples. **Results:** Hydroxyphenyllactic acid, argininosuccinic acid and norepinephrine were selected by both algorithms. The model reached an AUC of 0.954 in the training batch and 0.839 (95% CI 0.687–0.991) in the validation batch. In repeated cross-validation on all 65 samples, the AUC was 0.790 (95% CI 0.655–0.924), with sensitivity 0.714 and specificity 0.938; nested cross-validation gave 0.747. **Conclusions:** A three-metabolite CSF panel discriminates LM from non-LM and retains discrimination under cross-validation and in a temporally independent batch. It is a candidate adjunct to cytology and imaging whose specificity, based on 16 controls, requires confirmation in larger cohorts.

## 1. Introduction

Leptomeningeal metastasis (LM) is a common and severe complication of the central nervous system in patients with advanced malignancies, occurring in approximately 5% to 8% of those with solid tumors, most frequently in breast cancer, lung cancer, and melanoma [1,2]. The incidence of LM from solid tumors has been rising with the extended survival achieved by targeted and immunotherapies and the continuous improvement in diagnostic strategies; the prognosis remains extremely poor, with an expected survival of only 3–6 months after treatment [3]. Currently, the diagnosis of LM mainly relies on a comprehensive assessment of the patient’s history of malignancy, clinical manifestations, neuroimaging findings, and cerebrospinal fluid (CSF) cytology. However, the sensitivity and specificity of these diagnostic approaches remain suboptimal. Therefore, there is an urgent clinical need for more accurate molecular biomarkers to aid in diagnosis [4,5]. With the advancement of metabolomics, CSF, an essential biofluid that directly reflects the central nervous system microenvironment, has been recognized as an ideal source for identifying molecular biomarkers of LM [6]. Tumor development and progression are closely associated with metabolic reprogramming. The classical Warburg effect reveals that tumor cells tend to sustain rapid proliferation through aerobic glycolysis [7]. Beyond glucose metabolism, dysregulation of amino acid, lipid, and nucleotide metabolism also plays critical roles in tumor progression, immune evasion, and drug resistance [8,9,10]. For example, glutamine metabolism provides both nitrogen and carbon sources for tumor cells, supporting biosynthesis and redox balance [11]; tryptophan-derived kynurenine suppresses immune responses by activating the aryl hydrocarbon receptor (AhR) signaling pathway [12]; and disturbances in nucleotide metabolism directly affect DNA/RNA synthesis and genomic stability [13]. Recent untargeted metabolomic analyses of cerebrospinal fluid (CSF) from patients with non-small cell lung cancer (NSCLC)-associated LM revealed significant differences in multiple endogenous metabolites, such as tyrosine, pyruvate, and tryptophan, between LM and control groups, indicating that metabolic reprogramming involving amino acid metabolism and glycolysis is closely linked to LM pathophysiology [14]. Another targeted LC–MS study identified differential expression of lipid metabolites, amino acids, glycerol derivatives, and cholesterol esters between LM patients and healthy or primary brain tumor (PBT) controls, with several lipid markers demonstrating complete discriminative ability [15]. Collectively, these findings suggest that metabolic alterations are not merely secondary phenomena of tumor progression but may actively drive the onset and development of LM, providing valuable insights for understanding its molecular mechanisms and for developing novel diagnostic approaches.

In this context, omics approaches have increasingly become important tools for elucidating the biological mechanisms of LM. Boire and colleagues reported that complement C3 can remodel the CSF microenvironment, creating conditions that facilitate tumor cell colonization in the leptomeninges [16]. Proteomic studies have also revealed the diagnostic potential of specific proteins in CSF [17]. Compared with proteomics, metabolomics is particularly well-suited for exploring metabolic adaptation mechanisms in LM and identifying clinically translatable biomarkers, as it sensitively captures disturbances in metabolic pathways and directly reflects cellular functional states [18,19]. Preliminary studies have shown that CSF metabolic profiles detected by NMR and LC–MS can distinguish LM from non-LM patients and are closely associated with prognosis [14,20]. Therefore, CSF metabolomics not only reveals the metabolic reprogramming characteristics of LM but may also serve as a valuable tool for precision diagnosis and therapy.

In this study, targeted metabolomics was applied to the CSF of patients with and without LM, in combination with two machine learning algorithms, to identify key metabolites and construct a diagnostic model. The model was developed in one analytical batch and applied unchanged to a second, temporally independent batch, and was additionally evaluated by repeated and nested cross-validation on the whole cohort. The purpose of the study is to characterize the CSF metabolic features of LM, to propose a compact metabolite panel as a candidate adjunct to cytology and imaging, and to define the limits of what a single-center cohort of this size can establish.

## 2. Materials and Methods

### 2.1. Patients and Sample Collection

This study was conducted in accordance with the principles of the Declaration of Helsinki and the Belmont Report. CSF samples were collected after written informed consent was obtained from all participants. All patients included in this study were clinically suspected of leptomeningeal involvement and underwent lumbar puncture for CSF cytological evaluation. The CSF samples analyzed in this study were residual specimens collected after routine clinical testing. Exclusion criteria included the presence of central nervous system infection, autoimmune diseases, or recent systemic treatments such as radiotherapy or chemotherapy prior to sampling. The study protocol was approved by the Ethics Committee of the Cancer Hospital, Chinese Academy of Medical Sciences and Peking Union Medical College (approval number 23/090-3829, approved on 20 March 2023).

LM was diagnosed according to the EANO-ESMO criteria [3]. Confirmed LM required positive CSF cytology; probable LM required typical leptomeningeal enhancement on contrast-enhanced MRI together with compatible clinical findings in the presence of negative or suspicious cytology on repeated examinations. Patients were classified as non-LM only when three or more CSF cytological examinations, accumulated over a surveillance period exceeding six months, were negative, contrast-enhanced MRI of the brain (with spinal imaging in a subset of patients) showed no leptomeningeal enhancement, and no clinical sign attributable to leptomeningeal involvement was present. The sample entered into the analysis was that obtained at the examination at which leptomeningeal status was established. Clinical follow-up after sampling was continued for a median of 6.3 months (range 0.4–26.8) in the non-LM group, and no non-LM patient developed LM.

**Figure 1:**
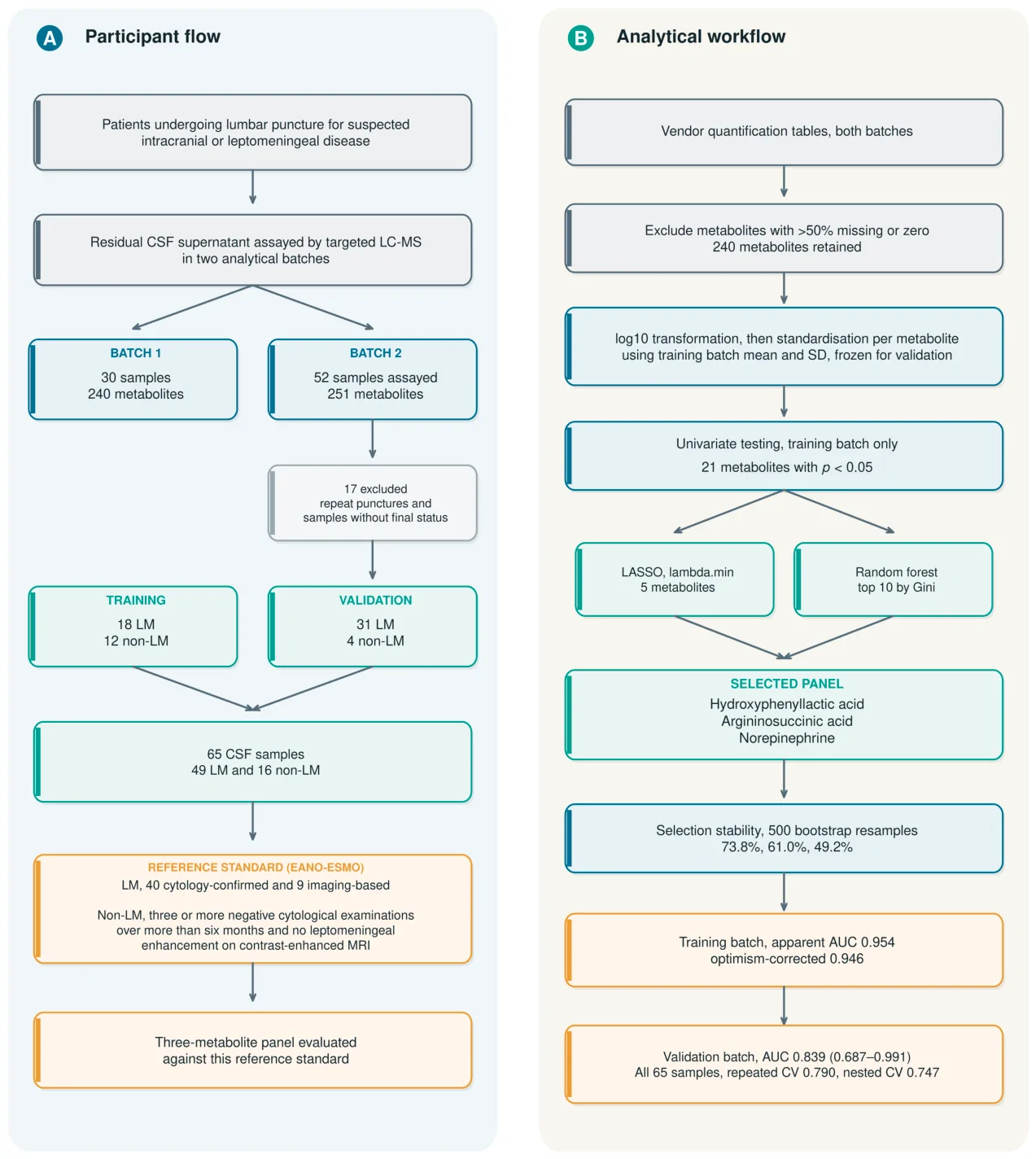
Participant flow (**A**) and analytical workflow (**B**). LM, leptomeningeal metastasis; LC–MS, liquid chromatography–mass spectrometry; AUC, area under the curve; CV, cross-validation.

A total of 65 CSF samples were analyzed: 49 from patients with LM (40 confirmed, 9 probable) and 16 from patients with intracranial neoplasms or other intracranial lesions without LM. Samples were assayed by targeted LC–MS in two analytical batches in chronological order of collection. The first batch (*n* = 30; 18 LM, 12 non-LM) served as the training batch. The second batch comprised 52 assayed samples, of which 17 were not entered into the analysis dataset: 11 were serial punctures from patients already represented by the puncture at which leptomeningeal status was established, and 6 came from patients who underwent a single puncture at our center and did not return, so that their leptomeningeal status could not be established. The remaining 35 samples (31 LM, 4 non-LM) formed a temporally independent validation batch. The participant flow is shown in Figure 1A.

CSF samples were obtained by lumbar puncture, immediately placed on ice after collection, and processed within half an hour. Following centrifugation to remove cellular components, the supernatant was aliquoted and stored at –80 °C until analysis.

### 2.2. LC–MS-Based Metabolomic Analysis

Metabolite extraction and detection were performed by Shanghai Applied Protein Technology Co., Ltd. (Shanghai, China), following previously described methods [21]. In brief, CSF samples were thawed on ice, and pre-cooled methanol/acetonitrile/water (2:2:1, *v*/*v*/*v*) was added. After thorough mixing, samples were sonicated in an ice-water bath for 30 min and incubated at –20 °C for 10 min. The mixtures were then centrifuged at 14,000 *g* and 4 °C for 20 min, and the supernatants were collected and dried under vacuum. The dried extracts were reconstituted in 100 μL of acetonitrile/water (1:1, *v*/*v*), centrifuged again (14,000 *g*, 4 °C, 15 min), and the resulting supernatants were subjected to LC–MS analysis. Liquid chromatography was performed using an Agilent 1290 Infinity UHPLC system (Agilent Technologies, Santa Clara, CA, USA) and AB Sciex QTRAP 6500+ mass spectrometer (SCIEX, Framingham, MA, USA); the columns are from Waters Corporation (Milford, MA, USA). Chromatographic separation was achieved using either an HILIC column (Waters BEH Amide, 2.1mm × 100 mm, 1.7 µm) or a C18 column (Waters BEH C18, 2.1mm × 100 mm, 1.7 µm). Data acquisition and quantitative analysis were conducted using MultiQuant 3.0.3 or Analyst 1.6.3 (SCIEX, Framingham, MA, USA). Quality control (QC) samples were analyzed alongside biological samples throughout the experiment, and metabolites with a coefficient of variation (CV) less than 30% in QC samples were considered reliably detected.

### 2.3. Data Preprocessing and Identification of Differential Metabolites

All analyses started from the vendor quantification tables of the two batches. Metabolites with more than 50% missing or zero values in either batch were excluded. Concentrations were log10-transformed and each metabolite was standardized to zero mean and unit variance across samples. For the batch-split analyses, the mean and standard deviation of the training batch were used, and the same frozen parameters were applied to the validation batch; for the pooled analyses each batch was standardized separately. Differential metabolites were identified in the training batch only. Normality was assessed with the Shapiro–Wilk test; normally distributed metabolites were compared by independent samples *t*-test and the remainder by the Mann–Whitney U test. Metabolites with *p* < 0.05 were defined as differential; fold change (LM relative to non-LM, on the original concentration scale) is reported descriptively. Differential metabolites were mapped to KEGG compound identifiers and tested for pathway enrichment against all detected and annotated metabolites using the hypergeometric test; pathways with *p* < 0.05 were reported. The rich factor of a pathway is the number of differential metabolites annotated to that pathway divided by the number of detected metabolites annotated to it.

### 2.4. Machine Learning-Based Feature Selection

Feature selection was confined to the training batch and used the differential metabolites as candidates. LASSO logistic regression was fitted with the glmnet package, with the penalty selected by 10-fold cross-validation (lambda.min). A random forest of 1000 trees was fitted with the randomForest package and metabolites were ranked by mean decrease in Gini impurity. Metabolites selected by LASSO and ranked within the top ten by random forest were defined as the panel. The stability of selection was quantified by repeating the whole procedure in 500 bootstrap resamples of the training batch and recording, for each metabolite, the proportion of resamples in which it was selected by both algorithms.

### 2.5. Construction of the Diagnostic Model

A logistic regression model with the panel metabolites as predictors was fitted in the training batch. Apparent discrimination was quantified by the area under the receiver operating characteristic curve (AUC) with DeLong confidence intervals (pROC package), and corrected for optimism by 1000 bootstrap resamples. A cut-off was defined by the Youden index in the training batch and held fixed thereafter. Sensitivity, specificity and predictive values are reported with Wilson confidence intervals.

### 2.6. Temporal Validation and Cross-Validation

The fitted model was applied without modification to the validation batch. Because the two batches were collected in different periods and analyzed in separate LC–MS runs at the same center, this constitutes temporal (internal–external) validation in the TRIPOD framework [22], not external validation. Because the validation batch contained only four non-LM samples, specificity cannot be estimated from it, and two further analyses were performed on all 65 samples so that all 16 controls contributed. First, the fixed three-metabolite model was evaluated by 10-fold cross-validation repeated 50 times, with out-of-fold predictions averaged across repeats. Second, a fully nested cross-validation was performed, in which univariate filtering, LASSO regression, random forest ranking and model fitting were all repeated inside every training fold of a 10-fold cross-validation repeated 20 times, so that the uncertainty of feature selection was charged against the estimate.

### 2.7. Nomogram, Calibration and Decision Curve Analysis

A nomogram was generated from the training-batch model with the rms package. Calibration was assessed by comparing predicted probabilities from the frozen model with observed proportions in the validation batch and in the cross-validated predictions, and summarized by the Brier score. Clinical utility was assessed by decision curve analysis (rmda package) using the frozen model’s predicted probabilities.

### 2.8. Risk Score and Subgroup Analysis

The predicted probability of the model served as the risk score. Risk scores were compared between LM and non-LM samples with the Wilcoxon rank-sum test in the training batch, in the validation batch and in the cross-validated predictions. Subgroup analyses used the cross-validated risk score and were stratified by age (median split), sex, glucose (<2.5 mmol/L), chloride (<120 mmol/L), white blood cell count (>8 × 10^6^/L), total cell count (median split) and analytical batch; ROC curves were plotted within each stratum.

### 2.9. Statistical Analysis and Software

All analyses were performed in R version 4.4.1 (glmnet 4.1, randomForest 4.7, pROC 1.18, rms 6.8, rmda 1.6). Continuous variables are given as mean ± standard deviation or median with interquartile range according to distribution and compared by *t*-test or Mann–Whitney U test; categorical variables are compared by the chi-square or Fisher exact test. Statistical significance was defined as *p* < 0.05. During the preparation of this manuscript, the authors used Claude Opus 5 (Anthropic) to improve the grammar, wording and readability of the English text; the tool was not used for study design, data processing, statistical analysis or interpretation, and the authors have reviewed and edited the output and take full responsibility for the content.

## 3. Results

### 3.1. Cohort Characteristics and Platform Stability

The training batch comprised 18 LM and 12 non-LM samples and the validation batch 31 LM and 4 non-LM samples (Table 1). The proportion of LM differed between batches (60.0% versus 88.6%, Fisher exact *p* = 0.010); age, sex, glucose, chloride, micro total protein, total cells and white blood cells did not differ. Of the 49 LM cases, 40 were cytologically confirmed and 9 were imaging-based probable LM (Table 2).

The QC correlation matrix showed correlation coefficients of 0.982 to 0.986 among QC samples (Figure S1A). Approximately 90% of detected peaks had a relative standard deviation below 30% (Figure S1B). In principal component analysis the QC samples clustered tightly and the biological samples showed no batch-related clustering (Figure S1C).

### 3.2. Differential Metabolites and Pathway Enrichment

**Figure 2:**
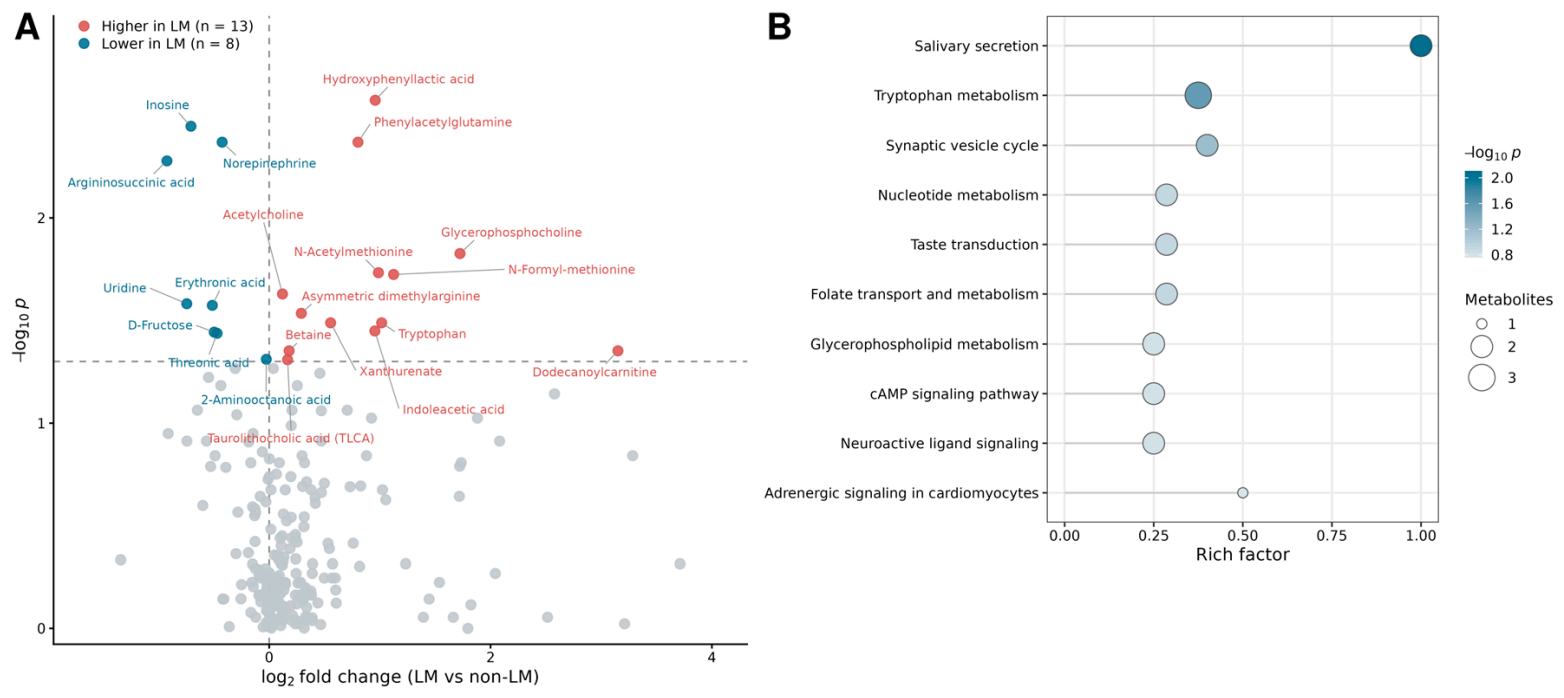
Differential metabolites in the training batch. (**A**) Volcano plot of 240 metabolites; the 21 metabolites with *p* < 0.05 are labeled, red higher and blue lower in LM. (**B**) KEGG pathways ranked by *p* value; point size gives the number of differential metabolites and color −log10 *p*; the rich factor is the number of differential metabolites annotated to a pathway divided by the number of detected metabolites annotated to it. In (A) the horizontal dashed line marks *p* = 0.05 and the vertical dashed line marks a log2 fold change of 0.

After filtering, 240 metabolites were available in the training batch (Table S1). Twenty-one differed significantly between LM and non-LM samples (*p* < 0.05): 13 were higher in LM, including hydroxyphenyllactic acid, phenylacetylglutamine, glycerophosphocholine, N-formyl-methionine, N-acetylmethionine, tryptophan, indoleacetic acid and dodecanoylcarnitine, and eight were lower, including argininosuccinic acid, norepinephrine, inosine and uridine (Figure 2A). Eleven of the 21 also showed a fold change greater than 1.5. Among KEGG metabolic pathways, tryptophan metabolism was enriched at *p* < 0.05 (xanthurenate, indoleacetic acid and tryptophan; rich factor 0.375, *p* = 0.027) (Figure 2B, Table S2).

### 3.3. Feature Selection

**Figure 3:**
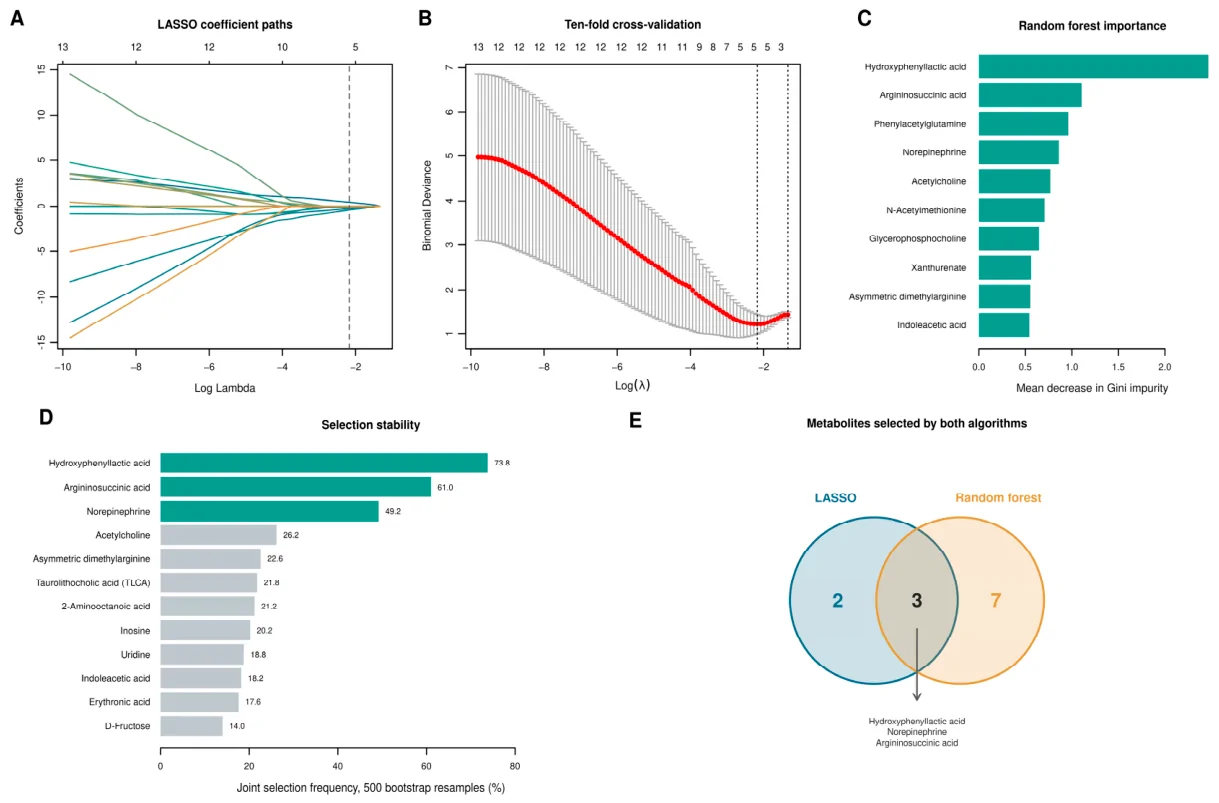
Feature selection in the training batch. (**A**) LASSO coefficient paths; each colored line is one candidate metabolite and the vertical dashed line marks lambda.min, the numbers along the top axis giving the number of non-zero coefficients. (**B**) Ten-fold cross-validation deviance against lambda; the red points are the mean binomial deviance, the grey bars its standard error, and the two dotted vertical lines mark lambda.min and lambda.1se. (**C**) Random forest variable importance (mean decrease in Gini impurity), top ten. (**D**) Joint selection frequency of each metabolite across 500 bootstrap resamples; green bars indicate the three metabolites selected by both algorithms. (**E**) Metabolites selected by both algorithms.

LASSO regression selected five of the 21 candidates (hydroxyphenyllactic acid, inosine, norepinephrine, argininosuccinic acid and uridine; Figure 3A,B, Table S3). Random forest ranked hydroxyphenyllactic acid, argininosuccinic acid, phenylacetylglutamine and norepinephrine highest (Figure 3C, Table S4). Three metabolites, hydroxyphenyllactic acid, argininosuccinic acid and norepinephrine, were selected by both algorithms (Figure 3E). In 500 bootstrap resamples of the training batch, these three were jointly selected in 73.8%, 61.0% and 49.2% of resamples respectively, the three highest values of any metabolite; no other metabolite exceeded 27% (Figure 3D, Table S5). Hydroxyphenyllactic acid was higher and argininosuccinic acid and norepinephrine lower in LM.

### 3.4. Model Construction, Temporal Validation and Cross-Validation

**Figure 4:**
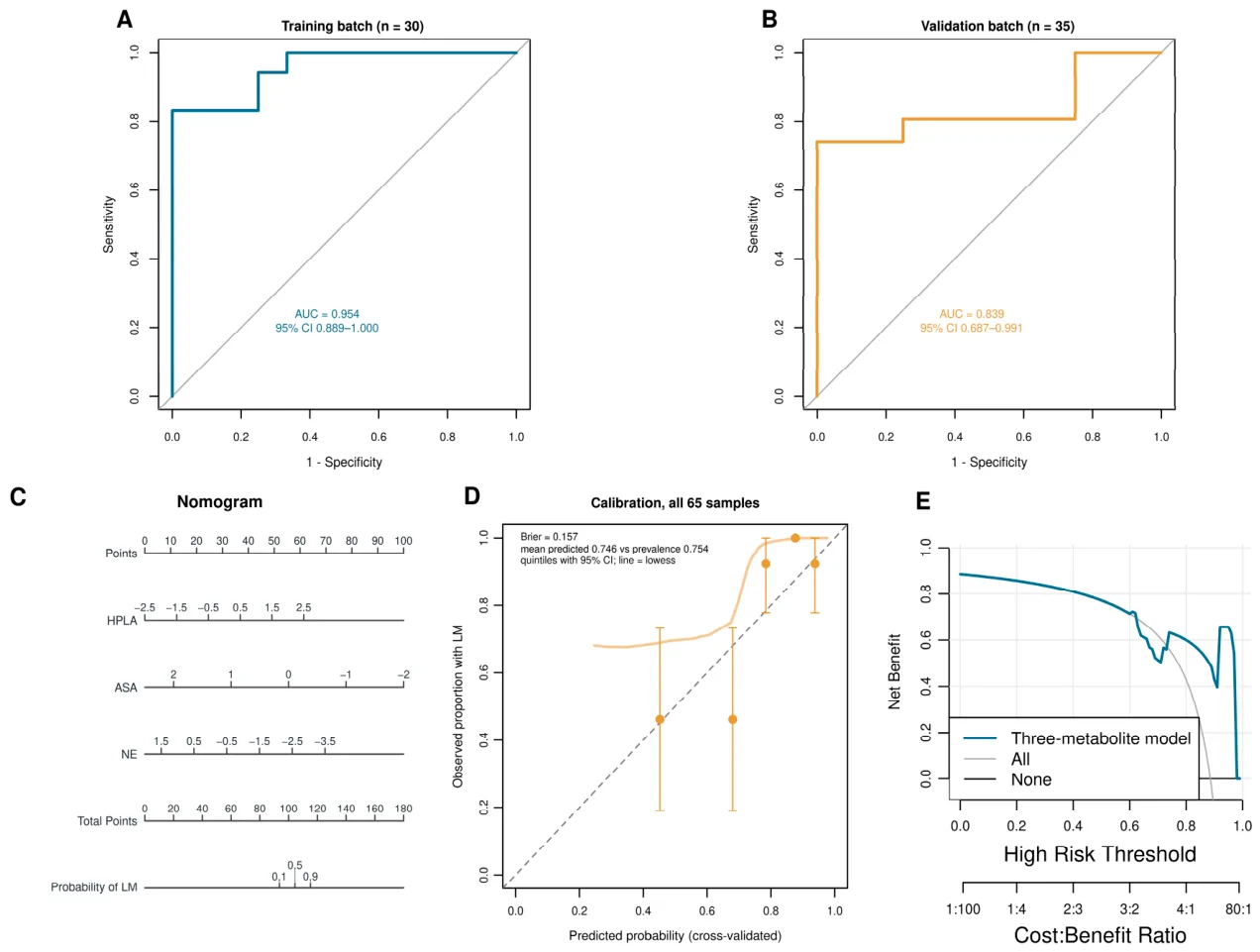
Diagnostic model. (**A**) ROC curve in the training batch (*n* = 30). (**B**) ROC curve of the frozen model in the validation batch (*n* = 35). (**C**) Nomogram. (**D**) Calibration of the cross-validated predictions on all 65 samples: observed proportion with LM against predicted probability in quintiles, with 95% confidence intervals; the line is a lowess smooth. (**E**) Decision curve analysis in the validation batch. In (**A**,**B**) the grey diagonal is the line of no discrimination, and in (**D**) the grey dashed diagonal indicates perfect calibration.

The logistic regression model fitted in the training batch was: logit(*p*) = 1.458 + 2.499 × [hydroxyphenyllactic acid] − 4.520 × [argininosuccinic acid] − 2.575 × [norepinephrine], with predictors as standardized log10 concentrations (Table 3). Its apparent AUC in the training batch was 0.954 (95% CI 0.889–1.000), 0.946 after optimism correction (Figure 4A). Applied unchanged to the validation batch, the model reached an AUC of 0.839 (95% CI 0.687–0.991) (Figure 4B). At the fixed cut-off of 0.702, sensitivity was 0.833 (95% CI 0.608–0.942) in the training batch and 0.742 (95% CI 0.568–0.863) in the validation batch, and specificity was 1.000 in both (95% CI 0.757–1.000 and 0.510–1.000).

In 10-fold cross-validation repeated 50 times on all 65 samples, the three-metabolite model reached an AUC of 0.790 (95% CI 0.655–0.924; standard deviation across repeats 0.021), with sensitivity 0.714, specificity 0.938, positive predictive value 0.972 and negative predictive value 0.517 at the Youden cut-off, all 16 controls contributing (Table 3). Fully nested cross-validation, in which feature selection was repeated inside every fold, gave an AUC of 0.747 (95% CI 0.606–0.889); norepinephrine was selected in 99.5% and hydroxyphenyllactic acid in 77.0% of the 200 folds. In the model refitted on all 65 samples, the coefficients were −1.391 for norepinephrine (*p* = 0.005), 0.753 for hydroxyphenyllactic acid (*p* = 0.096) and −0.495 for argininosuccinic acid (*p* = 0.223).

The nomogram is shown in Figure 4C. In the cross-validated predictions on all 65 samples the mean predicted probability was 0.746 against an observed prevalence of 0.754 (Brier score 0.157; Figure 4D); in the validation batch alone, the frozen model gave 0.708 against 0.886 (Brier score 0.177). In decision curve analysis the net benefit of the model exceeded that of the treat-all and treat-none strategies across intermediate threshold probabilities (Figure 4E).

When the LM group was restricted to cytologically confirmed cases, the AUC was 0.955 (95% CI 0.883–1.000) in the training batch (13 LM, 12 non-LM) and 0.870 (95% CI 0.733–1.000) in the validation batch (27 LM, 4 non-LM) (Table S6).

### 3.5. Risk Score Distribution

**Figure 5:**
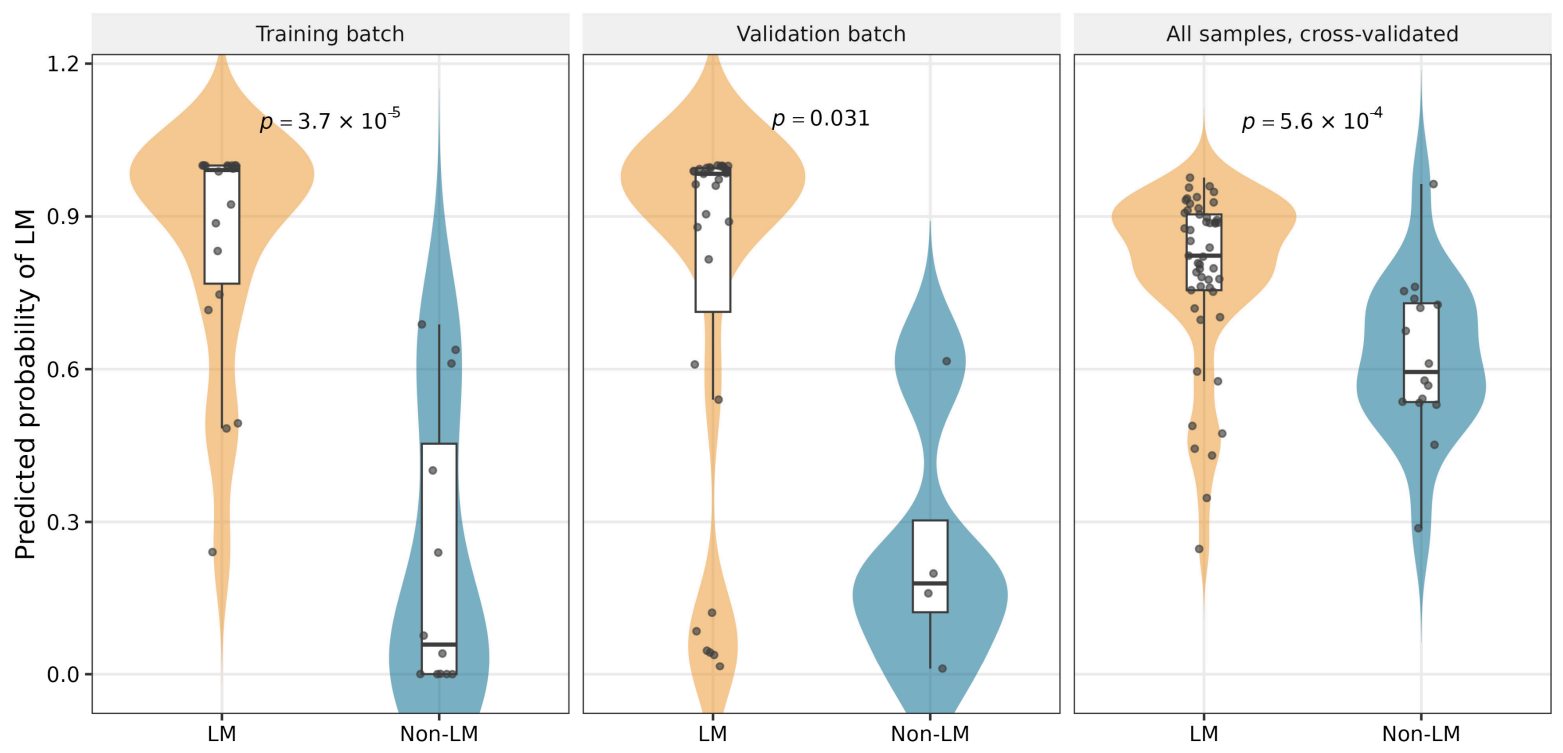
Predicted probability of LM by group in the training batch, the validation batch and the cross-validated predictions on all 65 samples; Wilcoxon rank-sum *p* values.

Risk scores were higher in LM than in non-LM samples in the training batch (Wilcoxon *p* = 3.7 × 10^−5^), in the validation batch (*p* = 0.031) and in the cross-validated predictions (*p* = 5.6 × 10^−4^) (Figure 5).

### 3.6. Subgroup Analysis

**Figure 6:**
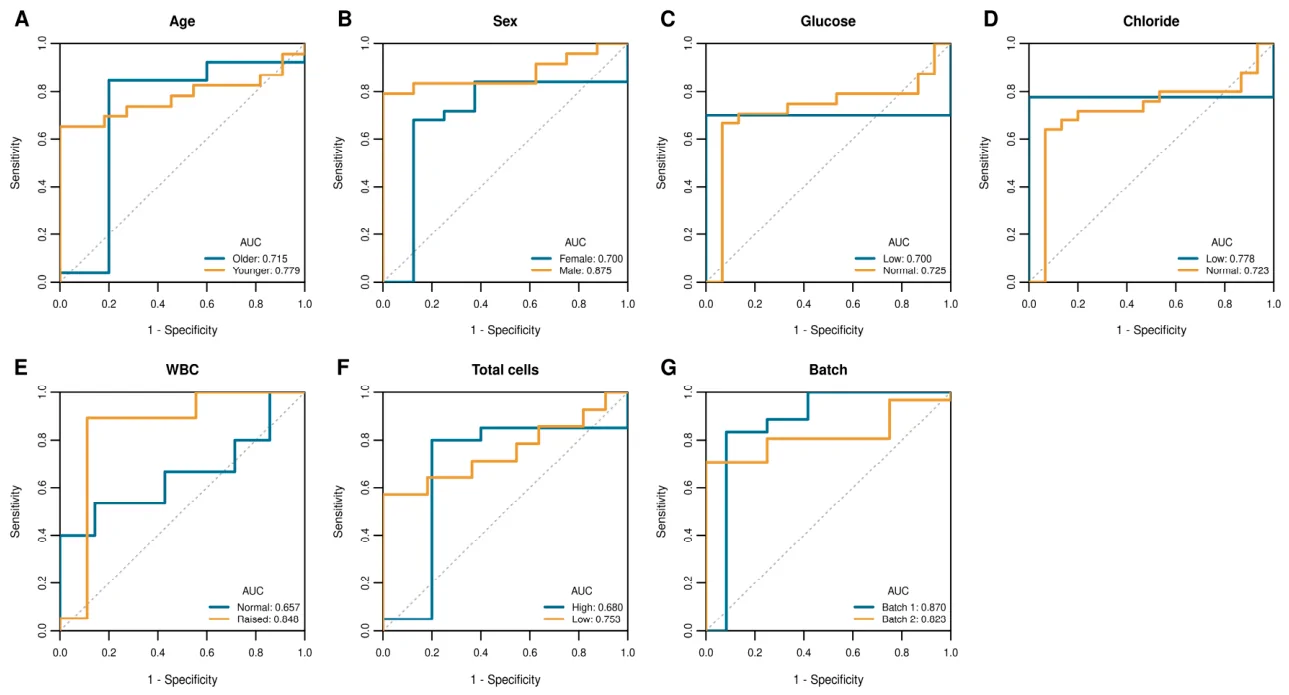
ROC curves of the cross-validated risk score within clinical subgroups: (**A**) age, (**B**) sex, (**C**) glucose, (**D**) chloride, (**E**) white blood cell count, (**F**) total cell count and (**G**) analytical batch. The grey dashed diagonal is the line of no discrimination.

Using the cross-validated risk score, the AUC within subgroups was: age (older 0.715, younger 0.779), sex (female 0.700, male 0.875), glucose (low 0.700, normal 0.725), chloride (low 0.778, normal 0.723), white blood cell count (normal 0.657, raised 0.848), total cell count (high 0.680, low 0.753) and analytical batch (batch 1 0.870, batch 2 0.823) (Figure 6, Table S7). Micro total protein was not analyzed as a subgroup because only two samples, both LM, had raised values.

## 4. Discussion

In this study, targeted metabolomic profiling of CSF combined with LASSO regression and random forest identified three metabolites, hydroxyphenyllactic acid, argininosuccinic acid and norepinephrine, that together discriminate LM from non-LM. The model reached an AUC of 0.954 in the training batch and 0.839 in a temporally independent batch, and 0.790 in repeated cross-validation on all 65 samples with all 16 controls contributing. The direction of the three metabolites was consistent across batches, the panel was the most stable under bootstrap resampling, and discrimination was unchanged when the LM group was restricted to cytologically confirmed cases.

The three metabolites point to two metabolic axes. Hydroxyphenyllactic acid and norepinephrine both derive from tyrosine, the former through transamination and the latter through the catecholamine pathway, and their opposite direction in LM is compatible with a shift in tyrosine handling within the leptomeningeal compartment; untargeted profiling of NSCLC-associated LM has likewise reported altered tyrosine [14]. Argininosuccinic acid is an intermediate of arginine biosynthesis, and its reduction in LM is compatible with altered arginine metabolism, which has been linked to tumor immune evasion [23,24,25,26,27]. We did not measure enzyme expression, enzyme activity or metabolic flux, and these interpretations are hypothesis-generating; any therapeutic inference would require experimental validation in cellular and animal models.

Norepinephrine requires particular caution. CSF catecholamine concentrations can be influenced by acute stress, neuroendocrine activation, sympathomimetic and psychotropic medication and the circumstances of sampling. Detailed medication histories were not systematically recorded in this retrospective cohort, so we cannot exclude that part of the norepinephrine signal reflects such factors rather than leptomeningeal disease. Norepinephrine was nevertheless the most stably selected metabolite in nested cross-validation and carried most of the discrimination in the full-cohort model, so it is retained in the panel; its behavior under controlled sampling conditions and with recorded medication is a priority for prospective work.

Compared with previous CSF metabolomic studies of LM [14,15,20], which reported broad alterations in amino acid and nucleotide metabolism, the present study adds a compact panel with a stated selection rule, an explicit estimate of selection stability, and evaluation in a second analytical batch. Rogachev et al. reported CSF metabolites with diagnostic or prognostic value in glioma [28], supporting the general premise that CSF metabolites carry disease-specific information.

From a clinical perspective, cytology remains the reference standard, with a sensitivity of approximately 70% on a single examination that rises with repeated sampling [4,5], and CSF cell-free DNA and circulating tumor cell assays are promising but complex and costly [29,30,31]. Metabolite profiling offers operational simplicity and minimal sample requirements [32,33]. The panel proposed here is not a stand-alone test and cannot replace cytology or imaging: a positive cytology is diagnostic and requires no further test, and with a cross-validated sensitivity of 0.714 and a negative predictive value of 0.517 the panel cannot be used to exclude LM. Its proposed role is in the frequent situation in which cytology is negative or equivocal while clinical suspicion persists. Because it uses the same residual CSF already obtained for routine analysis and requires no additional puncture, a high-risk score could support repeating the lumbar puncture, performing spinal MRI or initiating treatment earlier, whereas a low score would support continued observation. For context, a model built on six routine CSF chemistry parameters (glucose, chloride, micro total protein, total cells, white blood cells and adenosine deaminase) reached a cross-validated AUC of 0.648 in the same cohort. This proposed role is itself a hypothesis that must be tested prospectively with a predefined cut-off.

This study has limitations. First, the sample size is small and all samples came from a single center. Second, the two batches differ in the proportion of LM and the validation batch contains only four non-LM samples, so the batch-split analysis is a temporal check rather than an external validation and its specificity cannot be estimated; the cross-validated estimates on all 65 samples are therefore the primary figures. Third, the non-LM group consists of patients with intracranial neoplasms or other intracranial lesions, and the specificity of the panel against central nervous system infection, treatment-related meningeal reaction and parenchymal brain metastasis remains to be established. Fourth, LM was defined by a composite reference standard, although restriction to cytologically confirmed cases did not change the results. Fifth, no mechanistic experiments were performed and the pathway interpretation is hypothesis-generating. Multicenter prospective validation with an adequately sized and appropriately composed control group is the necessary next step.

## 5. Conclusions

Targeted CSF metabolomics identified a three-metabolite panel of hydroxyphenyllactic acid, argininosuccinic acid and norepinephrine that discriminates leptomeningeal metastasis from non-LM, with an AUC of 0.954 in the training batch, 0.839 in a temporally independent batch and 0.790 under cross-validation on the whole cohort. The panel is measured in residual CSF already obtained for routine testing and can complement cytology and imaging when cytology is negative or equivocal. Multicenter prospective studies are warranted to confirm its diagnostic value.

## Figures and Tables

**Table 1:** Baseline characteristics of the training and validation batches.

Characteristic	Training Batch (*n* = 30)	Validation Batch (*n* = 35)	*p*
LM, *n* (%)	18 (60.0)	31 (88.6)	0.010
Non-LM, *n* (%)	12 (40.0)	4 (11.4)	
Sex, male, *n* (%)	17 (56.7)	15 (42.9)	0.389
Age, years	48.97 ± 13.49	60.00 (41.00–67.00)	0.100
Glucose, mmol/L	3.36 ± 1.04	3.57 ± 1.50	0.577
Chloride, mmol/L	124.87 ± 6.53	124.99 ± 4.87	0.884
Micro total protein, mg/L	77.13 (44.94–131.27)	73.83 (40.15–122.28)	0.683
Total cells, ×10^6^/L	111.00 (11.00–238.75)	201.50 (103.50–327.00)	0.285
White blood cells, ×10^6^/L	12.00 (4.00–27.50)	6.50 (2.75–29.25)	0.382

Mean ± standard deviation with *t*-test, or median (interquartile range) with Mann–Whitney U test; categorical variables by chi-square or Fisher exact test. Routine CSF chemistry was unavailable for 4 training and 11 validation samples. LM, leptomeningeal metastasis.

**Table 2:** Diagnostic category of the 49 patients with LM according to the EANO-ESMO criteria.

Category	Training Batch (*n* = 18)	Validation Batch (*n* = 31)	Total
Confirmed LM	13	27	40
Probable LM	5	4	9

Confirmed LM, positive CSF cytology. Probable LM, typical leptomeningeal enhancement on contrast-enhanced MRI with compatible clinical findings and negative or suspicious cytology on repeated examinations.

**Table 3:** Diagnostic performance of the three-metabolite model.

Analysis	*n* (LM/non-LM)	AUC (95% CI)	Sensitivity (95% CI)	Specificity (95% CI)	PPV	NPV
Training batch	30 (18/12)	0.954 (0.889–1.000)	0.833 (0.608–0.942)	1.000 (0.757–1.000)	1.000	0.800
Training batch, optimism-corrected	30 (18/12)	0.946				
Validation batch	35 (31/4)	0.839 (0.687–0.991)	0.742 (0.568–0.863)	1.000 (0.510–1.000)	1.000	0.333
All 65 samples, repeated 10-fold cross-validation	65 (49/16)	0.790 (0.655–0.924)	0.714	0.938	0.972	0.517
All 65 samples, fully nested cross-validation	65 (49/16)	0.747 (0.606–0.889)				

Model: logit(*p*) = 1.458 + 2.499 × HPLA − 4.520 × ASA − 2.575 × NE (standardized log10 concentrations); cut-off 0.702 defined in the training batch. AUC confidence intervals by DeLong; sensitivity and specificity by Wilson.

## Data Availability

The metabolite quantification tables and the clinical variables used in the analysis are provided as Supplementary Materials; the analysis scripts and further data are available from the corresponding authors on reasonable request.

## Supplementary Materials

The following supporting information can be downloaded at: https://www.mdpi.com/article/10.3390/diagnostics16203316/s1, Figure S1: Quality control of the LC–MS platform: (A) correlation matrix of QC samples, (B) cumulative distribution of the relative standard deviation of detected peaks, (C) principal component analysis of QC and biological samples. Table S1: Differential analysis of all 240 metabolites in the training batch. Table S2: KEGG pathway enrichment of the 21 differential metabolites. Table S3: LASSO coefficients at lambda.min. Table S4: Random forest variable importance. Table S5: Selection stability across 500 bootstrap resamples. Table S6: Sensitivity analysis restricted to cytologically confirmed LM. Table S7: Subgroup analysis using the cross-validated risk score.

## Abbreviations

AhR	aryl hydrocarbon receptor
ASA	argininosuccinic acid
AUC	area under the curve
CSF	cerebrospinal fluid
CTCs	circulating tumor cells
ctDNA	circulating tumor DNA
CV	coefficient of variation
DCA	decision curve analysis
HPLA	hydroxyphenyllactic acid
NE	norepinephrine
LASSO	least absolute shrinkage and selection operator
LC-MS	liquid chromatography-mass spectrometry
LM	leptomeningeal metastasis
NSCLC	non-small cell lung cancer
NPV	negative predictive value
PCA	principal component analysis
QC	quality control
ROC	receiver operating characteristic
RSD	relative standard deviation
SD	standard deviation
PPV	positive predictive value
TRIPOD	Transparent Reporting of a multivariable prediction model for Individual Prognosis Or Diagnosis
EANO-ESMO	European Association of Neuro-Oncology–European Society for Medical Oncology
